# Assessment of Heavy Metal Contamination, Bioaccumulation, and Nutritional Quality in Fish from the Babina–Cernovca Romanian Sector of the Danube River

**DOI:** 10.3390/foods14193419

**Published:** 2025-10-03

**Authors:** Ioan Oroian, Bogdan Ioachim Bulete, Ecaterina Matei, Antonia Cristina Maria Odagiu, Petru Burduhos, Camelia Oroian, Ovidiu Daniel Ștefan, Daniela Bordea

**Affiliations:** 1 Faculty of Agriculture, University of Agricultural Sciences and Veterinary Medicine Cluj-Napoca, 3–5 Calea Mănăștur, 400372 Cluj-Napoca, Romania; neluoroian@yahoo.fr (I.O.); ovidiu.stefan@usamvcluj.ro (O.D.Ș.); daniela.bordea@usamvcluj.ro (D.B.); 2 Danube Delta Biosphere Reserve Administration, 34A Portului Street, 820243 Tulcea, Romania; Bbulete@ddbra.ro; 3 Faculty of Materials Science and Engineering, National University of Science and Technology Politehnica Bucharest, Splaiul Independenței no. 313, Sector 6, 060042 Bucharest, Romania; ecaterina.matei@upb.ro; 4 Faculty of Horticulture and Business in Rural Development, University of Agricultural Sciences and Veterinary Medicine Cluj-Napoca, 3–5 Calea Mănăștur, 400372 Cluj-Napoca, Romania

**Keywords:** bioconcentration factor, bio-sediment accumulation factor, habitat, proximate composition, muscular tissue

## Abstract

Danube Delta (DD), an ecologically vulnerable site, together with fish populations, which are significant food resources, are largely exposed to heavy metal contamination. This study was developed in the Babina–Cernovca sector of DD in September 2023. Zinc (Zn), and iron (Fe) were identified in water, while copper (Cu), iron (Fe), and manganese (Mn) were in sediments (mud). Proximate composition of the muscle tissues of eight fish species identified in the area was assessed. The muscle was also tested to identify heavy metals contamination. The contamination degree was assessed using bioaccumulation and bioconcentrations factors. The relation between nutritional parameters and metals was tested using bivariate and multivariate analyses. Samples were analyzed by specific laboratory tests, and data were processed using ANOVA, Spearman correlation, Principal Component Analysis (PCA), and hierarchical clustering. *S. erythrophthalmus*, *C. gibelio*, and *A. alburnus* have the highest metal bioaccumulation capacity, exhibiting species-specific accumulation patterns. PCA and clustering analysis reflect the influence of species and environmental factors on heavy metal accumulation in fish tissue. The study integrates the heavy metals content with nutritional parameters in fish muscular tissue, using bivariate and multivariate analysis for assessing fish vulnerability to heavy metals exposure in the Danube River.

## 1. Introduction

For several decades, global aquatic ecosystems have been under continuous pressure from anthropogenic activities, which has led to the accumulation of heavy metals in water, sediments, and biota, with serious ecological implications. Heavy metals such as copper (Cu), zinc (Zn), iron (Fe), and manganese (Mn) are essential for physiological processes among aquatic organisms, but in elevated levels disturb the biochemical pathways and the metabolic functions of the species, destabilizing the ecosystem’s integrity [1,2]. Cadmium (Cd), chromium (Cr), lead (Pb), mercury (Hg), and metalloid arsenic (As) have highly toxic potential. They are systemic toxic agents with harmful capacity for human health, even at low exposure. These elements are frequently considered carcinogenic agents [3,4,5,6].

Harmful runoffs as results of industrial growth, intensive agriculture inputs (mainly fertilizers and pesticides), and discharge of city waste have made a major contribution to increased input of heavy metals in freshwater ecosystems, which have high potential in disturbing their balance [7]. Their increased occurrence in natural specific environments may cause dramatic changes in present fauna, such as modifications of species compositions or affected reproductive functions, which could cause population declines, endangering even the species’ existence in such habitats [8]. The human population from the vicinity of such areas is also threatened by the presence of potential pollutants, because it depends on water resources for a large variety of immediate uses (fishing, drinking, washing, etc.) [9,10,11]. From being primary consumers to becoming predators, fish are relevant across many different trophic levels. Due to this reason, they have capacity to integrate exposure to heavy metals as result of interaction with their aqueous life environment and with sediments [12]. Based on this consideration, fish species are considered suitable biomonitoring agents (sentinels) for assessing heavy metal contamination of water resources [13,14,15]. The diversity of their habitat, and function of species peculiarities, which may differ from benthic to pelagic, allows the identification of spatial variability of metal distributions across the aquatic ecosystem [16,17]. The fish muscle tissue is considered the most important because it is an appreciated component of human food, and it must be taken into consideration that it represents an appropriate route for the toxic heavy metals (Hg, Cd, Pb, or metalloid As) transfer into the food chain of [12,13,15,16,17]. Heavy metals, once ingested by fish, can interact with their muscle proximate composition represented by parameters such as protein, fat, ash, or nitrogen-free matter. Testing their level of accumulation in fish tissue results in valuable information concerning biochemical mechanisms and metabolic functions (energy storage, enzyme function, cellular mechanisms, etc.) associated with metal accumulation. Metals can bind proteins, induce oxidative stress, or induce disorders in lipid metabolism. By these mechanisms, they may influence physiological homeostasis and interfere with vital processes such as growth, and/or reproduction [1,6,18]. Some fish species have special affinity for heavy metal bioconcentration. For example, investigations conducted by Hristov et al. (2024) in Biosphere Reserve “Srebarna,” Bulgaria, showed high amounts of lead in *Scardinius erythrophthalmus* L. and *Ceratophyllum demersum* L. bodies, from sources, besides feed, such as the water and sediments, which emphasizes their sensitivity to environmental pollution [19].

Based on fishes’ capacity for heavy metal bioconcentration and the implication of their high potential to be used as environmental bioindicators for indexing heavy metal contamination status in aquatic ecosystems, their role in establishing the pollution level, mainly in protected sites of ecosystemic relevance, is peremptory. Such an example is the Danube Delta, a UNESCO Biosphere Reserve and a RAMSAR wetland, which is of singular ecological importance. At present, besides climate change’s specific challenges, this area faces heavy metal accumulation problems, induced by anthropogenic activities that occur upstream on the shores of nine intersecting countries [20,21,22,23]. The studies on heavy metal contamination in the Danube Delta mostly focus on certain contaminants, and do not integrate data across environmental matrices and biological compartments. Very few investigations have simultaneously assessed heavy metal concentrations in water, sediments, and fish muscle tissue, their relationships with proximate nutritional composition, and species-specific bioaccumulation potential using advanced multivariate statistics. To our knowledge, no published work has combined these components in the Babina–Cernovca sector of the Romanian Danube Delta, leaving a critical gap in understanding the complex interactions between metal contamination, fish nutritional quality, and potential risks for human consumption.

The present study aims to an integrate approach of heavy metal pollution in a site-specific area by conducting a research aiming to: (i) Identify and quantify essential, non-essential and toxic heavy metals in water, sediment, and muscle tissue of eight fish species from the Babina–Cernovca sector of the Romanian Danube Delta; (ii) assess proximate composition parameters of fish muscle and explore their relationships with heavy metal content; (iii) identifying the bioaccumulation capacity for each species and sampling point; (iv) identifying patterns of metal association, species clustering, and potential bioindicator species, and (v) tracking the differences in the accumulation of heavy metals among fish species with distinct feeding behaviors. Beyond its ecological and biomonitoring relevance, the study also addresses food safety implications by evaluating whether detected concentrations in edible fish muscles comply with internationally recognized maximum residue limits. In particular, the focus on toxic metals such as Cd and Hg provides critical evidence for assessing consumer health risks, given that fish represents a significant dietary protein source for local populations. This dual perspective ensures that the findings are not only useful for understanding ecosystem health and species-specific bioaccumulation pathways but also directly applicable to public health protection and sustainable fishery management in the Danube Delta.

## 2. Materials and Methods

### 2.1. Location

**Figure 1:**
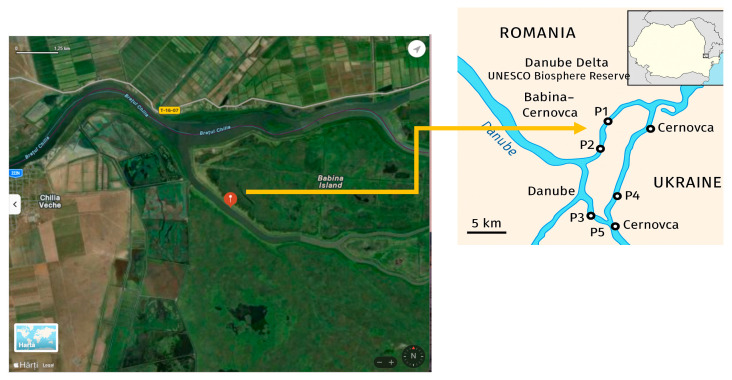
Geographical location of the Babina–Cernovca Romanian sector of the Danube Delta (45.4141159 N; 29.3774357 E) and the sampling points for water, mud, and fish. Harta [In Romanian]—map; Hărți [In Romanian]—maps.

Sampling was conducted in the Babina–Cernovca sector of the Romanian Danube Delta (Figure 1). The Danube Delta is characterized by interconnected channels, floodplains, and shallow lakes. Five sampling points (P1–P5) indicate the sites where water, surface mud (0–5 cm), and fish species were collected in September 2023 for heavy metal and proximate composition analyses.

A mobile GPS was use to record geographic coordinates. The site selection was performed based on representativeness of habitat and its accessibility. Sampling took place in September 2023.

GPS and site selection were based on accessibility and representativeness of local habitat conditions.

### 2.2. Sampling

**Water and sediments**. The water samples (in 5 replicates) were collected from each sampling point in polyethylene bottles. They were stored at 4 °C until deliverance to laboratory. Sediment (mud) samples (0–5 cm depth; *n* = 5 replicates) were collected using an Ekman grab sampler. After collecting, they were placed in acid-washed polyethylene containers, maintained on ice, and transported to the laboratory.

**Fish species**. Forty adult freshwater fish individuals of comparable size (five exemplars by species) were sampled with nets (Table 1). Fish were euthanized according to ethical guidelines stipulated by Romanian regulations, muscle tissue was collected and stored at −20 °C prior to analysis [24,25,26].

### 2.3. Laboratory Analysis

**Proximate composition**. Dry matter (DM) was determined via gravimetric method by oven-drying at 105 °C to constant weight. Protein content was measured by the Kjeldahl method (N × 6.25). Fat was determined via Soxhlet extraction with petroleum ether. Ash was measured by incineration in a muffle furnace at 550 °C. Nitrogen-free matter (NFM) was calculated by difference [28]. All samples were analyzed in 2 replicates.

**Heavy metals determination**. The determination of heavy metal from river water and mud were performed by Atomic Absorption, Flame Technique with ContrAA 800 equipment, Analytik Jena (AG Germany, Hanover, Germany). ICP multielement standard solution IV (1000 mg/L in HNO_3_, Merck KGaA, Darmstadt, Germany) was used for calibration. The analysis of heavy metals in fish muscle tissues was conducted using Energy Dispersive X-ray Fluorescence Spectrometry (EDXRF), employing an Elvatech spectrometer (Elvatech Ltd., Kyiv, Ukraine)). Prior to measurement, tissue samples were lyophilized to achieve a constant dry mass, homogenized into fine powders, and pressed into analytical-grade pellets. Ti, Fe, Zn, Cr, Cu, Sb, Mn, Cd, Ag, Ni, Se, V, Sn, Hg, and metalloid As were identified. All samples were analyzed in 2 replicates. We have to mention that concerning both AAS and XRF analyses, we did not use certified reference materials. For this reason, for water samples (AAS), we performed the calibration with high-purity standard solutions and we used repeated measurements to verify the analytical reliability. The XRF calibration was made taking ino account the fundamental parameters of the method and varification compared with internally characterized samples. In both cases, this resulted in low standard errors (±0.001–0.007), confirming in this way the accuracy of determinations.

### 2.4. Bioconcentration Factor (BCF) and Biota-Sediment Accumulation Factor (BSAF)

The BCF [29] and BSAF [30] for each metal were calculated separately for water and sediment as:

(1) $$BCF=\frac{Metal\;concentration\;in\;fish\;muscle\;(mg/kg)}{Metal\;concentration\;in\;water,\;\;\;dissolved\;(mg/L)}L/kg$$

(2) $$BSAF=\frac{Metal\;concentration\;in\;fish\;muscle\;(mg/kg)}{Metal\;concentration\;in\;sediment\;(mg/kg)}$$

To facilitate the hierarchization of species function of bioaccumulation heavy metals capacity, we propose the integration of BCF and BSAF, according to relation

(3) $$\sum {log}_{10}BCF+\sum {Log}_{10}BSAF$$

BCF values were determined for Zn and Fe from water, and BSAF values for Cu, Zn, Fe, and Mn from sediments in all species at each sampling point. Where a metal was below detection, BCF, and BSAF were recorded as zero. Concentrations of biota and in surrounding environment are expressed as a ratio in both BCF and BSAF, the corresponding values often vary over several orders of magnitude. For this reason, to normalize data and enable their comparability, the logarithmic transformation (log_10_) of the BCF and BSAF [31] are used. There are no regulated thresholds for log BCF or log BSAF for metals as there are for some organic contaminants, but benchmarks are in widespread use [32].

### 2.5. Statistics

Statistical analyses were performed in XLSTAT (Addinsoft, Paris, France, version 2024.1). One-way ANOVA was applied to test differences between heavy metal concentrations in water, and sediment (*p* < 0.05) from sampling points. Spearman correlations were used to evaluate relationships between measured variables [33]. Hierarchical clustering (Ward’s method, Euclidean distance) was used to identify species groups with similar metal accumulation profiles [34]. Principal Component Analysis (PCA) was employed to explore multivariate relationships; for fish muscle data, the first two components (PC1 and PC2). For testing the opportunity of implementing factorial approach (Principal Components Analysis), the Bartlett (*p* < 0.01) and Keiser–Meyer–Olkin (KMO) tests (at threshold value above 0.500) were applied [33,34].

### 2.6. Ethical Considerations

Fish sampling and handling complied with Romanian national legislation and the guidelines of the Danube Delta Biosphere Reserve Authority. Necessary research permits were obtained prior to fieldwork (ARBDD-316/05.09.2023) [26].

## 3. Results

### 3.1. The Heavy Metals Concetration in Water and Mud in the Samplig Area

In water samples, only two heavy metals were detected, Zn (0.126 ± 0.002 mg/L m) in sampling point P4, and Fe (1.04 × 10^−6^ ± 2.63 × 10^−8^) in trace in sampling point P3 (Figure 1, Table 2). Iron was also detected in sample P4. The Zn concentration at the sampling point places the water from the sampling point in the second-class surface water quality, according to Romanian regulations [35].

**Figure 2:**
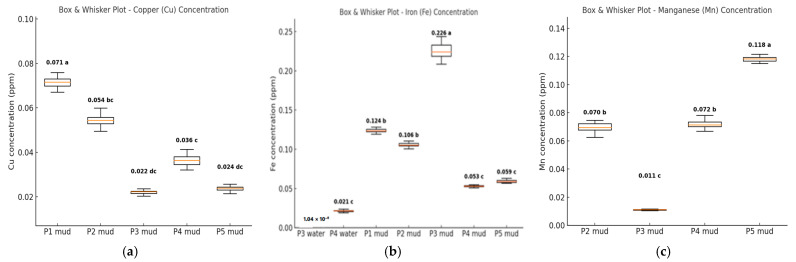
The box plots of the heavy metals (Cu, Fe, Mn) concentrations (mg/L) in water and mud from Babina–Cernovca sector of the Danube Delta (Mean ± SE). (**a**) Cu concentration (mg/kg) in mud samples; (**b**) Fe concentration (mg/kg) in water and mud samples; (**c**) Mn concentration (mg/kg) in mud samples; the differences between any two averages are significant, if their values are followed by different letters, or groups of different letters (*p* < 0.05).

In mud, Cu average concentrations exhibit a spatial gradient from P1 to P3 (0.022 mg/L). The average Cu concentrations in mud are much lower compared to Romanian standards for sediment chemical quality [35]. The statistical approach shows that Cu amounts determined in P1 (0.071 mg/L) and P2 (0.054 mg/L) sampling points are significantly higher than those quantified in P3–P5 sampling points, with P4 (0.036 mg/L) and P5 (0.024 mg/L) showing intermediate, yet still reduced, values (Table 1, Figure 2a).

A different situation is observed for Fe and Mn, which are the other two heavy metals determined in mud. They show greater heterogeneity in their spatial distribution compared with Cu but exhibit opposite spatial trends. The lowest average Fe content (0.053 ppm) was observed at sampling point P4 and the highest (0.226 mg/L) at P3 (Table 1). As shown in the box plot diagram (Figure 2b), Fe, largely associated with mud rather than with water, had a significantly higher average in P3 compared with those measured in P1 (0.124 ± 00.003 mg/L), P2 (0.106 ± 0.002 mg/L), P4 (0.053 ± 0.001 mg/L), and P5 (0.059 ± 0.002 mg/L) in mud, as well as in P3 and P4 in water.

Mn was observed in mud at only four sampling points, with average concentrations ranging from 0.011 ± 0.35 × 10^−3^ mg/L at P3 to 0.118 ± 0.002 mg/L at P5 (Table 1).

The maximum Mn average concentration (P5, 0.118 ± 0.002 mg/L) is statistically different from those observed in P2 (0.070 ± 0.003 mg/L), P3 (0.011 ± 0.35 × 10^−3^ mg/L), and P4 (0.072 ± 0.003 mg/L) (Figure 2c). All measured heavy metals have low standard errors, showing a uniform spatial distribution in mud regardless of sampling points (Table 2, Figure 2), with values ranging from ±0.001 (mg/kg) to ±0.009 (mg/kg).

### 3.2. The Proximate Composition and Heavy Metals in the Muscle Tissue of the Fish Species

The nutritional profile and heavy metal content of muscular tissue of eight fish species sampled from the Babina–Cernovca sector of the Danube Delta in September 2023 have distinctive characteristics (Table 3 and Table 4). *S. erythrophthalmus* and *C. carpio* are the fish species that have the highest average dry matter (30.20 ± 1.75% and 29.94 ± 1.60%, respectively), while the lowest content is observed in *A. alburnus* (22.47%).

**Proximate composition.** The highest protein contents (%DM) were seen in *C. gibelio* (79.25 ± 1.26) and *A. alburnus* (77.93 ± 1.4), while the lowest was observed in *C. carpio* (55.81 ± 1.62). Fat average content in *C. carpio* is over six-fold higher (11.36 ± 0.31) compared to *A. alburnus* average fat (1.87 ± 0.03). Contrarily, species such as *E. lucius* and *A. aspius* have leaner tissue profiles. Ash, which represents the total minerals content, ranges from 3.87 ± 0.06% in *S. erythrophthalmus*, and up to 6.10 ± 0.14% in *A. brama*. For the average content of nitrogen-free extracts (glycogen, other soluble carbohydrates, etc.), the highest values of 29.87 ± 1.11% and 27.76 ± 1.84% were observed in *S. erythrophthalmus* and *C. carpio*, respectively (Table 4).

**Heavy metals.** Ti levels ranged within the interval 0.19 ± 0.009 mg/kg in *C. gibelio*–0.30 ± 0.011 mg/kg in *A. alburnus*, with no statistically significant differences among studied fish species. Fe corresponds to the widest concentrations range, from 0.18 ± 0.006 mg/kg in *L. idus* to nearly ten-fold higher levels in *A. alburnus* (2 ± 0.048 mg/kg). Significantly lower Zn averages were observed in *A. aspius* and *L. idus*. Zn content ranged from 0.10 ± 0.003 mg/kg (*A. brama*) to 0.20 ± 0.005 mg/kg (*S. erythrophthalmus*), with significant differences between species.

In *A. aspius* Cr was not detected, in *L. idus* it has the highest average, while in *C. carpio* (0.51 ± 0.016 mg/kg), it has the lowest. Cu average concentrations range within a narrow interval (0.26 ± 0.016–0.31 ± 0.017 mg/kg). Sb was not detected in *E. lucius* and *C. gibelio* but in *A. brama*, where it was quantified in an amount of 0.31 ± 0.007 mg/kg, while concentrations in the other species were with an order of magnitude lower. Mn was detected in low concentrations only in *S. erythrophthalmus* (0.02 ± 0.002 mg/kg), *C. gibelio* (0.03 ± 0.002 mg/kg), and *A. alburnus* (0.02 ± 0.004 mg/kg). Cd levels range from 0.14 ± (0.005–0.007) mg/kg in *S. erythrophthalmus*, *A. aspius* and *C. carpio* to 0.25 ± 0.005 mg/kg in *A. alburnus*. It was observed in all eight fish species, with concentrations over three- to five-fold and the maximum limit (0.05 mg/kg) [35]. Ag, Ni, and Sn were detected at low concentrations, with no statistically significant differences among species. Se concentrations range from 0.99 ± 0.012 mg/kg in *S. erythrophthalmus* and 3.16 ± 0.094 mg/kg in *A. aspius*. V, Hg and As were detected in few species and at low levels. V was detected in *A. aspius* and *C. gibelio* in the same concentration (0.05 ± 0.002 mg/kg), As only in *E. lucius* (2.04 ± 0.045 mg/kg), *L. idus* (2.05 ± 0.062 mg/kg), *C. carpio* (2.07 ± 0.076 mg/kg), and *A. brama* (1.99 ± 0.097 mg/kg), in very similar concentrations, and Hg only *A. brama* (0.07 ± 0.004 mg/kg).

**Figure 3:**
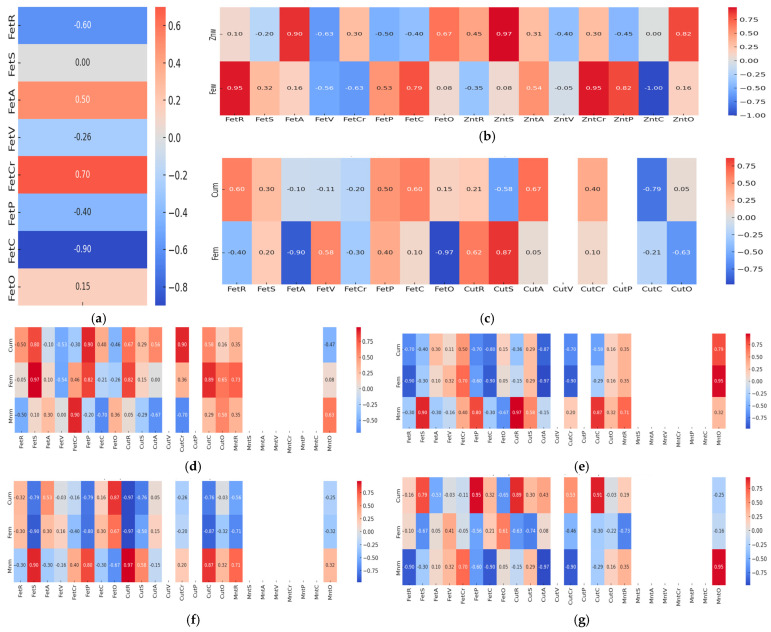
The Spearman correlations between heavy metals identified in water, mud and water, mud, and muscle tissue of eight fish species from the Babina–Cernovca sector of Danube Delta. (**a**) Fe from water collected from P3 location; (**b**) Zn and Fe from water collected from P4 location; (**c**) Cu and Fe from mud collected from P1 location; (**d**) Cu, Fe and Mn from mud collected from P2 location; (**e**) Cu, Fe, and Mn from mud collected from P3 location; (**f**) Cu, Fe, and Mn from mud collected from P4 location; (**g**) Cu, Fe and Mn from mud collected from P5 location; R-*Scardinius erythrophthalmus* L.; S-*Esox lucius* L.; A-*Aspius aspius* L.; V-*Leuciscus idus* L.; Cr-*Cyprinus carpio* P; f-*Abramis brama* L.; C-*Carassius gibelio* Bloch; O-*Alburnus alburnus* L.

The relationships between heavy metal concentrations detected in environmental matrices (water and mud) and their accumulation in the muscle tissue of eight fish species are emphasized using simple Spearman correlations (Figure 3). The correlation profile of Fe concentrations from water in P3 and fish tissue is dominated by a strong negative association in *C. gibelio* (R_S_ = −0.90), positive moderate (R_S_ = 0.50), and moderate to strong (R_S_ = 0.70) in *A. aspius* and *C. carpio*, respectively (Figure 3a). Zn from water sampled from P4 is very strongly correlated with Zn from muscle tissue of *L. idus* (R_S_ = 0.97) and *A. brama* (R_S_ = 0.95) species (Figure 3b). Fe sampled from water in the same point is also very strongly correlated with Fe from *S. erythrophthalmus* muscular tissue (R_S_ = 0.95) and strongly with *C. carpio* (R_S_ = 0.82) tissue (Figure 3b). In P1, mud copper concentration is positively moderate and strongly correlated with copper from *E. lucius* (R_S_ = 0.67) and *C. carpio* (R_S_ = 0.87), respectively, whereas Fe is strongly negatively associated with Fe from *C. gibelio* (R_S_ = −0.79) muscle tissue (Figure 3c). In P2, mud manganese concentration is positively moderately and very strongly correlated with manganese from *C. carpio* (R_S_ = 0.97) and *A. brama* muscle tissues (R_S_ = 0.95). Mud copper is also very strongly correlated with Cu from muscle tissue of *S. erythrophthalmus* (R_S_ = 0.90).

Iron from P2 mud is weakly correlated with iron from fish muscle tissues (Figure 3d). In P3, Mn from mud is very strongly positively correlated with Mn from muscle tissue of *C. carpio* (R_S_ = 0.97), while Fe is moderately-to-strongly positively correlated with Fe from muscle tissue of *S. erythrophthalmus* (R_S_ = 0.95). Cu from mud is moderately correlated with Cu from fish muscle tissues (Figure 3e).

In P4, Cu and Fe from mud are strongly correlated with their concentrations in fish tissue of *C. carpio* (R_S_ = 0.90), and *Sc. erythrophthalmus* (R_S_ = 0.89), respectively. Mn from mud is moderately correlated with fish muscle tissues in all species (Figure 3f). In P5, Mn from mud is positively very strongly correlated with Mn from muscle tissue of *A. alburnus* (R_S_ = 0.95). Cu and Fe from mud are moderately correlated with their concentrations in fish muscle tissues (Figure 3g).

**Figure 4:**
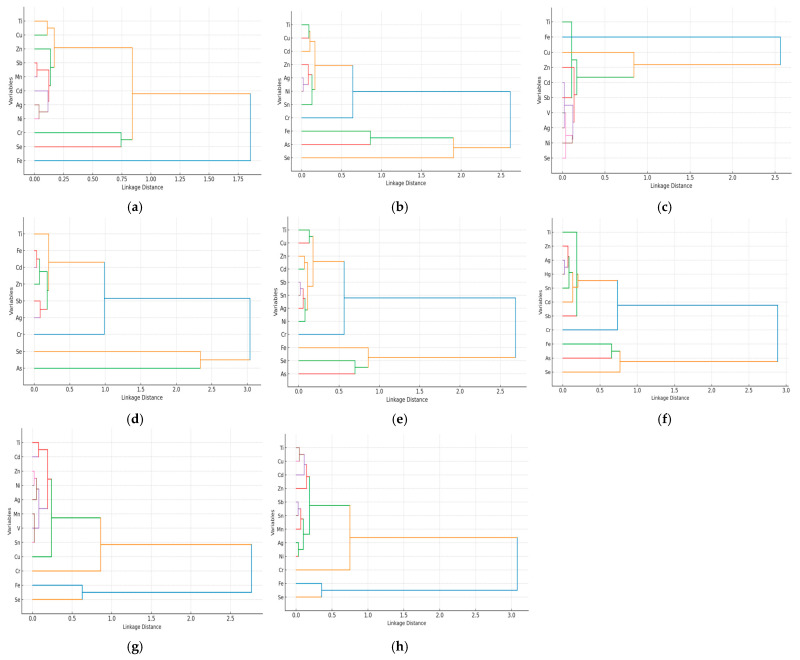
Hierarchical clustering of heavy metals in the muscle tissue of fish species from the Babina–Cernovca sector of the Danube Delta. (**a**) *Scardinius erythrophthalmus* L.; (**b**) *Esox lucius* L.; (**c**) *Aspius aspius* L.; (**d**) *Leuciscus idus* L.; (**e**) *Cyprinus carpio* P; (**f**) *Abramis brama* L.; (**g**) *Carassius gibelio* Bloch; (**h**) *Alburnus alburnus* L.

The dendrograms emphasize the species-specific association patterns of heavy metals bioaccumulated in fish muscle tissue (Figure 4). We observed two association patterns in all eight fish species studied.

One concerns frequent clustering of Fe with Se, and sometimes with As in *E. lucius* (Figure 4b), *C. carpio* (Figure 4e), *A. brama* (Figure 4f), *C. gibelio* (Figure 4g), and *A. alburnus* (Figure 4h).

The other concerns the clustering of Ti and Cu in *S. erythrophthalmus*, *L. idus* (Figure 4a), *E. lucius* (Figure 4b), *C. carpio* (Figure 4e), *C. gibelio* (Figure 4g), and *A. alburnus* (Figure 4h). In water samples because Fe concentration in P4 were very low, BCF cannot be calculated. The highest BCF for Fe is observed in *S. erythrophthalmus* and *A. alburnus*, while also for Zn in the same species *A. alburnus*, and for *C. gibelio*, with most species showing high BCF above 8 for Zn, and above 1 for Fe (Table 5).

### 3.3. Bioconcentration Factor (BCF) and Biota-Sediment Accumulation Factor (BSAF) Identified in the Fish Muscle Tissue

In mud, Fe BSAF was calculated in all sampling points. For Cu, BASF was calculated for six species, while for Mn only for *S. erythrophthalmus*, *C. gibelio*, and *A. alburnus* (Table 6, Table 7 and Table 8). BSAF values for Cu ranged in the interval 7.66–9.14 (log_10_ BSAF frames within 0.84–0.92) in species where it was identified (Table 6).

For iron, BASF values ranged within a very large interval of 23.09–2.08 (log_10_ BSAF frames within 0.84–0.92) in species where it was identified (Table 7). For Mn, BSAF could be calculated only for *S. erythrophthalmus*, *C. gibelio*, and *A. alburnus*, all below 1.00, indicating similarity in biota-sediment accumulation capability of all three species (Table 8).

### 3.4. Correlation and Multivariate Analysis of Heavy Metals Bioaccumulation and Proximate Composition in Fish Muscle Tissue

**Figure 5:**
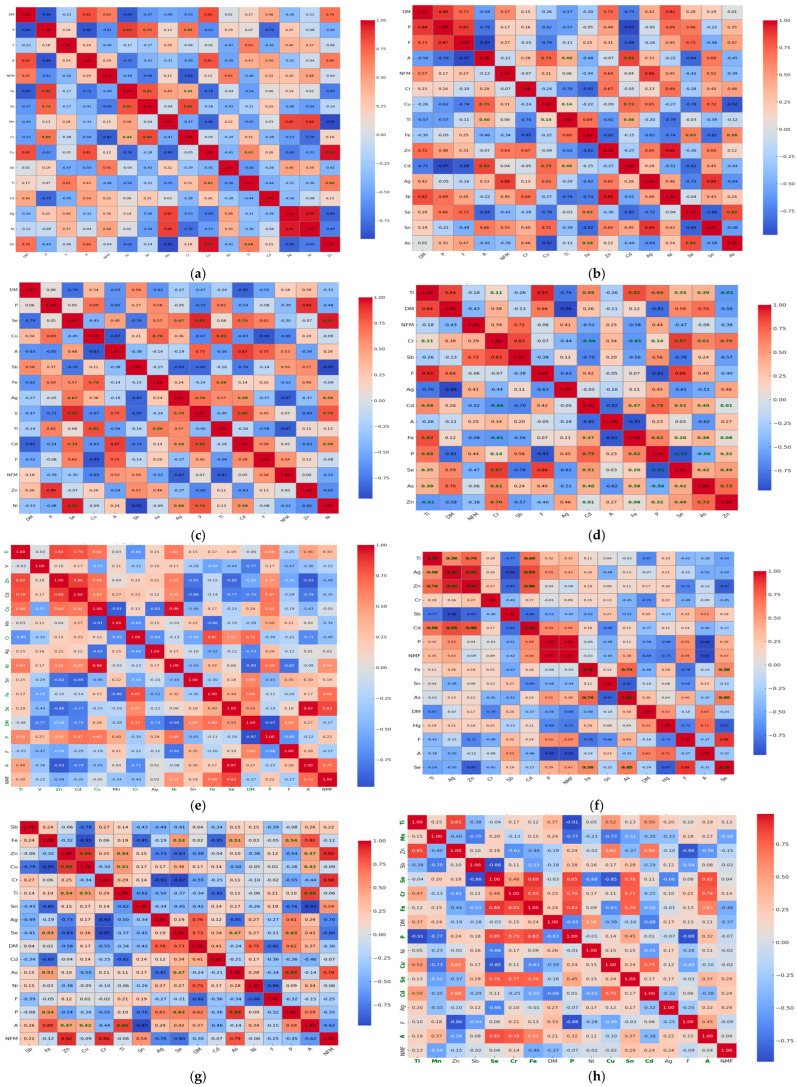
Spearman correlations between heavy metals and proximate composition of muscle tissue of fish species from the Babina–Cernovca sector of the Danube Delta. (**a**) *Scardinius erythrophthalmus* L.; (**b**) *Esox lucius* L.; (**c**) *Aspius aspius* L.; (**d**) *Leuciscus idus* L.; (**e**) *Cyprinus carpio* L.; (**f**) *Abramis brama* L.; (**g**) *Carassius gibelio* Bloch; (**h**) *Alburnus alburnus* L.

The correlation analysis reveals distinct, species-specific relationships between heavy metals (Figure 5). Across species, positive correlations between metals such as Fe, Se, and As and protein content are mainly in *S. erythrophthalmus* and *C. gibelio*, where protein levels show strong, positive associations with Se and Fe.

**Figure 6:**
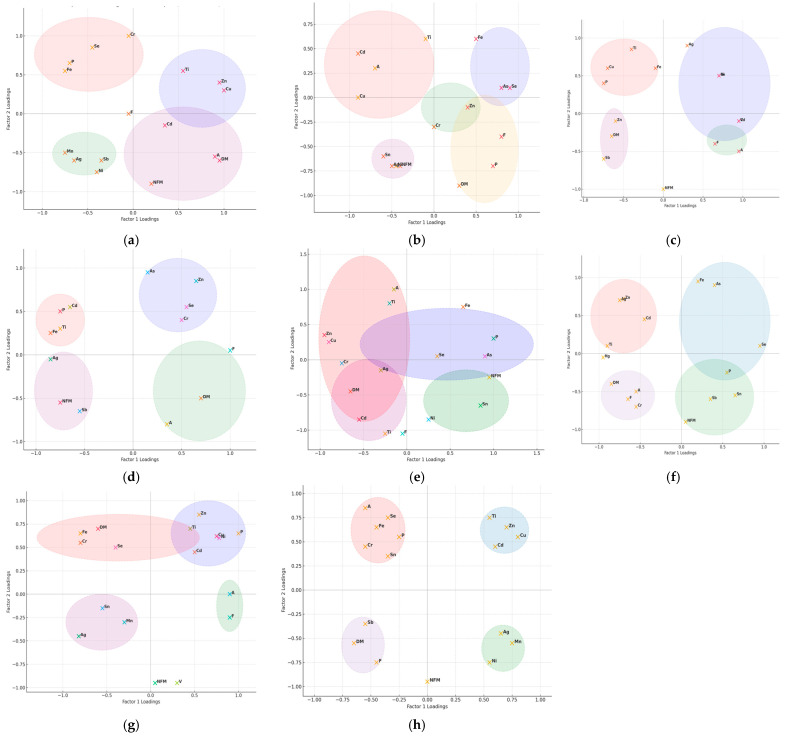
Principal Component Analysis (PCA) biplots showing the distribution of proximate composition variables and heavy metals in the muscle tissue of fish species from the Babina–Cernovca sector of the Danube Delta (PC1 × PC2). (**a**) *Scardinius erythrophthalmus* L.; (**b**) *Esox lucius* L.; (**c**) *Aspius aspius* L.; (**d**) *Leuciscus idus* L.; (**e**) *Cyprinus carpio* P; (**f**) *Abramis brama* L.; (**g**) *Carassius gibelio* Bloch; (**h**) *Alburnus alburnus* L. The colors are used for emphasizing the spatial segregation of variables, and overlapping of colors has no significance.

In some species like *E. lucius*, *A. aspius*, *A. brama*, *C. carpio* and *A. Alburnus*, we observe negative correlations between Zn or Cu and lipid fractions, while in the majority of species, high Fe and Se levels correlate negatively with nitrogen-free extract (NFM). The ash content correlates positively with Cd in *S. erythrophthalmus*, *E. lucius*, and *A. aspius* (Figure 5). The PCA captures the multivariate structure of the datasets, with the first two principal components explaining approximately 70.72–78.85% of total variance (Figure 6, Table 9).

The principal factors identified are fish species (F1), fish living environment (F2), feed (F3), heavy metals occurrence in sampling environment (F4). To simplify the grouping criteria, we discuss the biplot analysis as representation of F1 fish species (PC1) and F2 environmental conditions (PC2).

The clear spatial segregation of variables into distinct ellipses (Figure 6) indicates strong correlations and potential co-association patterns between heavy metals and proximate composition parameters, function of fish species, and or the heavy metal occurrence, confirmed by the intensity of the Spearman correlations (Figure 5). Dry matter (SU) and protein (P) are clustered and in majority of cases are positively associated with fish species. Their grouping may reflect biochemical processes related to organic matter. In some of the species studied, it is observed that there is a tendency of no association between nitrogen-free matters (NFM) and other nutrients or metals (Figure 6c,g,h). The clustering patterns of heavy metals give information about feed input sources, accumulation routes, or heavy metals metabolic roles [22]. The grouping of heavy metals usually suggests anthropogenic inputs mixed with environmental sources.

The biplot representation shows that titanium, zinc, iron, selenium, cadmium, copper, and nickel grouped in majority of fish species, and most influenced by environmental factors are the heavy metals most influenced by environmental factors. It also shows that nutritional factors are grouped and most influenced by fish species.

## 4. Discussion

### 4.1. The Heavy Metals Concetration in Water and Mud from the Samplig Area

In the Babina–Cernovca sampling sector of Danube Delta, sediments serve as the primary repository for some heavy metals (Cu, Fe, Mn), with water-phase concentrations below detection limits during the summer sampling period. Spatial heterogeneity of detected metals distribution function as sampling points, with sediments consistently acting as the dominant reservoir compared to the water environment. We consider that these variations are the result of environmental gradients produced as a consequence of sampling points particularities (Figure 2).

In a study conducted by Catianis et al. (2022) surface waters from 25 lakes in the Romanian Danube Delta between 2019 and 2021 were analyzed. They identified Cd, Co, Cr, Cu, Ni, Pb, and Zn [36], while we found only Fe (low concentrations in P3 and P4), and Zn (126 μg/L in P4). Compared with Romanian national legislation [35], which classifies the water in quality in five classes (from very good to poor), the Zn concentration we identified frames within II^nd^ class (good). It slightly exceeds the U.S. EPA’s aquatic life threshold of 120 μg/L [37]. It falls within EU’s Environmental Quality Standards (EQS), 3.1–1300 μg/L [38,39,40]. Studies performed in the Danube River and its lakes show a large variety of information in this area (Table 10).

Tudor et al. (2016) reported Zn concentrations in the Danube River and five Danube Delta lakes over a 15-year period (Table 10), and some of their values are comparable to our findings [41]. Milanov et al. (2016) found on the Danube River upstream of Belgrade found Fe and Zn concentrations (Table 10), which allow the framing of the river water as quality class II according to Serbian legislation [42].

A review of Danube River water quality on Romanian territory from 2010 up to 2021, elaborated by Cordeli (Savescu) et al. (2023), emphasizes from literature sources [43] (Table 10) Fe concentrations in river water similar to our findings [44], but also in much higher amounts [45] and a very large interval of concentrations for Zn [46,47]. In the lower Danube Delta, Simionov et al. (2021) (Table 10), found higher Fe, but much lower Zn concentrations, compared to our results [48]. Poleksic et al. (2010) analyzed water from two Serbian sectors of the Danube River (Table 10), and found higher Fe concentrations compared to our findings, while Zn concentrations were comparable to our results [49]. Milanov et al. (2016) identified bigger amounts of Fe and Cu in sediments from Danube River (Table 10), compared with our findings [42]. They did not identify manganese, while we identified it in four of five sampling locations [42]. A review by Cordeli (Savescu) et al. (2023) of Danube River sediment quality on Romanian territory from 1950 to 2022 [43] (Table 10), reported much higher Cu and Fe concentrations compared with our study. Poleksic et al. (2010) analyzing sediments from two Serbian sectors of the Danube River (Table 10), found much higher Fe, Cu, and Mn concentrations compared with our study [49].

According to the literature, the results of our research shows that in the Babina–Cernovca sector of Danube River, we detected fewer metals, and in lower amounts compared with other studies, which we consider may be the consequence of regional differences in their accumulation.

**Table 10:** Comparative heavy metal concentrations in water (mg/L) and mud (mg/kg) from sampling points located in the Babina–Cernovca sector of the Danube Delta, and data from the literature.

Environment	Metal	Current Data (mg/L)	Literature Values (mg/L)	References
Water				
River	Fe	1.04 × 10^−6^; 0.021	0.330; 0.080; 1244.700; 1.41–0.33	[42,44,45,49]
Delta		-	1.330; 0.428	[48]
River	Zn	0.126	0.025–0.118; 0.032; 0.009, 333.78; 0.094–1.46	[41,42,44,46,49]
Delta		-	0.020; 0.002	[48]
Lakes		-	0.020–0.120	-
Sediment				
	Fe	0.053–0.226	16,104; 15.36; 17,530; 32,720–33,480	[42,47,49,50]
	Mn	0.011–0.118	1147–1359	-
	Cu	0.024–0.071	35.92; 2; 56; 21–26	[42,49,51,52]

### 4.2. The Proximate Composition and Heavy Metals from the Muscle Tissue

The proximate composition of muscle from eight fish species in the Babina–Cernovca area of the Romanian Danube Delta, collected during September 2023, shows interspecific variability in terms of protein, fat, ash, and nitrogen-free matter (NFM), reflecting ecological and physiological differences. Protein content differences between fish species are the result of dissimilarities regarding specific muscles developed and metabolic characteristics. Some species, after reaching maturity, accumulate significantly higher lipid levels, which might reflect such adaptations for energy storage. NFM variations indicate differences in metabolic adaptations for energy storage. The moderately variable total mineral composition emphasized by ash content may be the result of the differences between bone structures, or mineral availability in the environment of Danube Delta. Compared with proximate composition identified by Ljubojevic et al. (2013) in fish species from Danube River, we obtained similar results [53]. For example, in *E. lucius* they found 79% water, 18.43% protein, 1.61% fat, and 0.64% ash, while we quantified 75.48% water, 18.94% protein, 1.55% fat, and 1.35% ash. In other species, similarities are even higher. Thus, in *A. aspius*, 78.51% vs. 75.86% water, 18.07% vs. 18.18% protein, 2.78% vs. 2.22% fat, and 1.16% vs. 0.99% ash. In *C. carpio*, 73.73% vs. 70.06% water, 16.69% vs. 16.71% protein, 7.13% vs. 6.28% fat, and 0.88% vs. 1.52% ash. In *A. brama*, 78.66% vs. 73.13% water, 17.59% vs. 17.64% protein, 3.24% vs. 1.47% fat, and 0.80% vs. 1.64% ash. In most cases compared with Ljubojevic et al. (2013) findings, the reduced fat content identified in our species is associated with an increase in ash content [53].

Non-toxic metals show different variability patterns in fish muscle tissue. Ti, Ag, and Ni have a low uniform concentration across different species, suggesting uniform environmental exposure and a limited bioaccumulation gradient. Cu absence from two species indicates selective uptake or localized environmental factors limiting its availability. Fe and Zn, both essential for metabolic and immune functions, show the highest variability, with lower levels in some species, whereas Se, toxic at increased levels, also shows significant variation with one species distinctly lower, which could be attributed to a hyper-variable intake in diet or environmental availability. Sb, Mn, V, and Sn are detected only in some cases, indicating limited accumulation or low environmental presence. Such variability in concentrations may result from the fact that some of these species potentially face localized exposure to specific metal sources in the Danube Delta.

In *C*. *gibelio* (Table 11) we identified lower Fe, and much lower Ni and Zn content compared with other studies [54]. The Cd levels we identified in muscle tissue is 3–5 times above the safe threshold of 0.05 mg/kg [38,55,56], exceeding the values reported in other studies in the area [43,54,57], but similar with those reported by Gati et al. (2013) nearby Delta channels [58]. We determine Cr and Cu averages, lower than other reports [38,46,59]. In our study we did not identify Hg and As, while other studies emphasize their presence in these species [38,43,46,57,59,60].

In *A. brama* (Table 11), a wider variation in Fe content is reported in the literature [61,62], compared with our findings. In the literature, Cu [44,60] and Ni [44] are mentioned, while we did not identify these metals in our samples. We found Zn, which is much lower compared to concentrations reported by other studies [44,62]. The Cr and Cd occurrence emphasized by our studies was higher, and much higher, respectively, than reported by other studies [44,60,61]. We identified As and Hg in amounts identified in other studies [44,57,61,63].

In *C. carpio* (Table 11), we determined an Fe concentration ranging within intervals mentioned by other authors [42,60,61,62]. Ni and Zn occurrence in our samples was much lower compared with values reported by other studies [44,62,63,64,65,66]. Literature reports copper occurrence in fish muscle tissue [43,59], but we did not determine Cu in our samples. The review of Danube River water quality on Romanian territory, elaborated by Cordeli (Savescu) et al. (2023) [43], emphasizes from literature sources in much lower Cd occurrence in muscular tissue of *C. carpio* [42,44,63,64,66] compared with our level, which is 10 times over the magnitude order admitted by regulations [38,55,56]. Milanov et al. (2016) also identified in *C. carpio* muscular tissue an amount similar with ours [42]. We found Cr levels within interval cited by others [44,61,64,65], and the same for Cu [63,65], and As levels [42,53,57,61,63,65]. Literature mentions the Hg occurrence in *C. carpio* muscular tissue, but we did not identify its presence [42,63,67].

In *E. lucius* (Table 11), we quantified concentrations of Fe, Ni, and Zn, which are much higher than those mentioned by other findings for Fe [65] and Zn [63,64], and lower for Ni [43]. Cu concentrations in muscle cited by literature [64,65] are higher compared with our findings. We found much higher Cd concentrations compared with other findings [57,63]. Literature mentions Cr values within an interval which includes the concentration we determined in our study [64]. We did not identify Hg in fish muscle, but we found As. Other studies emphasize the presence of both in this species, [57,60,63].

Shukerova et al. (2017) in a study performed on the Bulgarian part of Danube River report cadmium concentrations of mg/kg in *A. alburnus* lower with ten orders of magnitude compared with our findings, but Gati et al. (2013) found the same order of magnitude for Cd concentrations in a study performed in Romanian Danube Delta channels in 2012 [58,61] (Table 11). Toxic metals Cd, Hg, metalloid As, and potentially toxic metals at high concentrations such as Cr and Se have distinct patterns in fish muscle tissues. Cd, with potential for causing kidney damage, is 3–5 times higher compared with the allowable limit (0.05 ppm) in all species. Its occurrence in all fish species suggests extensive environmental exposure within the area of study. Hg, a highly toxic metal, is found below the allowable limit (0.05 ppm) in only one species, indicating less contamination in the region. As it occurs in just four species, indicating selective bioaccumulation linked to habitat-specific exposure such as sediment-bound As in bottom-feeding species.

**Table 11:** Comparative heavy metal concentrations (mg/kg, wet weight) in fish muscle.

Species	Metal	Current Data (mg/kg)	Literature Values (mg/kg)	References
*C*. *gibelio*	Fe	1.71	7.25–8.05	[59]
	Ni	0.10	1.994–2.246	[54]
	Zn	0.11	10.26; 11.72	[54,59]
	Cd	0.20	0.017; 0.057–0.061; 0.057; 0.13	[43,54,57,58]
	Cr	0.69	1.208; 1.345	[38,43,60]
	Cu	0.31	0.715; 1.500	[38,43,60]
	Hg	-	0.025; 0.30; 0.094; 0.139	[38,43,45,59]
	As	-	0.031–0.604; 0.139–0.172	[38,57]
*A. brama*	Fe	1.79	1.31; 27.64	[61,62]
	Ni	-	0.02–1.05	[44]
	Zn	0.10	3.15–23.84	[44,62]
	Cd	0.15	0.004; 0.027	[44,60]
	Cr	0.61	0.20; 0.33	[44,61]
	Cu	-	0.14–1.49	[44,60]
	Hg	0.07	0.08; 0.121; 0.237	[44,57,63]
	As	1.99	0.035; 1.73	[57,61]
*C. carpio*	Fe	1.71	7.42–19.62; 1.31; 27.64; 0.008	[42,60,61,62]
	Ni	0.09	1.685; 0.02–1.05; 6.16; 59.01	[44,63,64,67]
	Zn	0.15	3.15; 23.84	[44,62]
	Cd	0.14	0.140; 0.005; 0.084	[42,43,44,63,64,66]
	Cr	0.51	0.010; 1.143; 0.20; 0.33	[44,61,63,65]
	Cu	0.26	0.14;1.49; 0.688; 5.100	[44,60]
	Hg	-	0.207; 0.393–0.466; 0.890	[42,63,67]
	As	2.07	0.01; 0.08; 0.66; 0.035; 1.73	[42,53,57,61,65,66]
*E. lucius*	Fe	1.71	9.97–10.10	[65]
	Ni	0.09	0.02–0.05	[44]
	Zn	0.13	5.10; 23.90	[63,64]
	Cd	0.20	0.015–0.044	[57,63]
	Cr	0.55	0.781–2.071	[64]
	Cu	0.28	0.548; 2.90	[64,65]
	Hg	-	0.021–0.025; 0.30; 0.094; 0.139; 0.236	[43,45,51,54,63]
	As	2.04	0.030–0.604; 0.139–0.172; 1.129	[57,59,63]
*A. alburnus*	Cd	0.25	0.046; 0.13	[58,61]

Cr and Se, though toxic at high concentrations, are present in safe limits in almost all fish species. Thus, the results show that differences in metal concentrations across species express differences in function of species and their peculiarities, and habitat preferences within the aquatic ecosystem, potentially impacted by anthropic inputs. The differences between species concerning Fe and Zn content are most likely related to their habits, from feeding to habitat preferences, but also to metabolic specificity. *A. aspius* and *L. idus* being predatory species, may accumulate lower Fe quantities due to differences in diet and trophic level. *S. erythrophthalmus* tend to accumulate higher Zn quantities, because bioavailability of this element is higher in benthic zones from where they feed. Also, physiological traits and environment may cause variations in metal uptake and storage in tissues. The results of our study are consistent with literature data concerning fish species from Danube River, which emphasize the prevalent presence of Fe, Zn, Cd in most common fish species in the area, with Cd levels often similar with those obtained in our study in *C. carpio*, exceeding at least 3-fold the allowed limits [48]. The large variation in the intensity of correlations between heavy metals from fish species and from water/mud function of sampling points suggests complex interactions between environmental conditions and bioaccumulation processes, and that they are species-specific (Figure 3). Strong and moderate positive associations which we identified in water and mud indicate that certain fish species may act as effective bioindicators, reflecting the presence of metals like iron, zinc, copper, and manganese in their habitat, which could signal potential pollution. Negative correlations, particularly with iron in some species, suggest physiological mechanisms or environmental factors that may possibly limit metal uptake. For example, in *C. carpio*, a strong correlation is identified between Fe from water and fish muscle (R = 0.70) in sampling point P3, while in P4, the correlation is moderately negative (R = −0.63), but between Fe from mud and muscle results in positive correlations in both points, with R = 0.70 in P3 (Figure 3e), and R = 0.46 in P3 (Figure 3f). We consider that the negative correlation identified in P3 may be the result of less Fe bioavailability, perhaps bound in insoluble complexes due to water–matter interactions specific to the Danube River.

The Fe associations with Se in fish species (*S. erythrophthalmus*, *C. gibelio*, and *A. alburnus*) and sometimes with As, particularly in *E. lucius*, *C. carpio*, and *A. brama*, suggest their common biochemical roles such as redox regulation or incorporation into metalloprotein complexes are possibly due to their common occurrence in feed. Cu and Ti (*S. erythrophthalmus*, *E. lucius*, *C. carpio*, and *A. alburnus*), or Cd and Zn clustering in muscle in the great majority of species studied indicates communal exposure pathways to both dietary and/or sedimentary sources. Differences in cluster patterns across species emphasize that they may depend on the interactions between the environmental metal distributions, trophic levels, and physiological traits. For example, in *A. aspius* and *A. Alburnus*, we observe broader, less cohesive clusters, which suggest different exposure routes. Combined correlation and clustering analyses show that while environmental sources drive metal common occurrence, also in terms of availability, species-specific traits shape their assimilation and storage in tissues (Figure 3 and Figure 4).

### 4.3. Bioconcentration Factor (BCF) and Biota-Sediment Accumulation Factor (BSAF) Identified in the Fish Muscle Tissue

**Figure 7:**
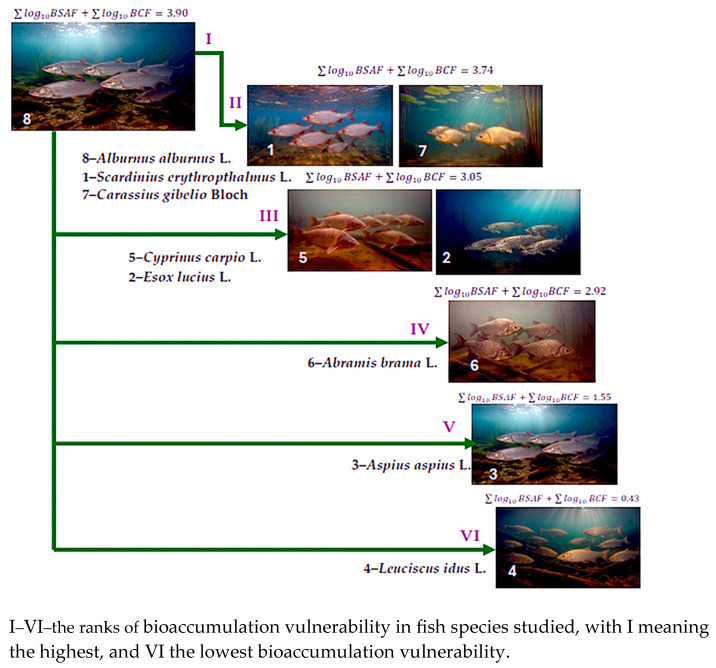
Integrated bioaccumulation vulnerability ranks in fish species in Babina–Cernovca sector of the Danube Delta.

The Fe highest bioconcentration (BCF, log_10_ BCF) is observed in *S. erythrophthalmus*, *E. lucius*, *C. carpio*, *A. brama*, *C. gibelio*, and *A. alburnus* but with low values and variations (1.03–1.59 kg/L for BCF, and 0.028–0.1790 for log_10_ BCF). For *A. brama* and *C. gibelio* they have subunitary BCF value. The BSAF trend is similar with that in sampling sites, but the order of accumulation was BSAF_P4_ > BSAF_P5_ > BSAF_P2_ > BSAF_P1_ > BSAF_P3_. *A. aspius* and *L. idus* have greater BSAF values while *A. brama* and *C. gibelio* have lower BCF. We consider that differences between the species with different lower BCF and BSAF, also in the same sampling point, and function of metal matrix (low BCF in *A. brama* and *C. gibelio* vs. BSAF in *A. aspius* and *L. idus*), may be the result of interaction between species-specific physiological mechanisms and metal environmental availability from habitat. Zn BCF and log_10_ BCF follow the same patterns as Fe BSAF but with higher values (8.14–9.52 kg/L BCF, 0.9106–0.9786log_10_ BSAF). Cu bioaccumulation increases significantly across sampling points, in the following order: BSAF_P3_ > BSAF_P5_ > BSAF_P4_ > BSAF_P2_ > BSAF_P1_. Most species show similar BSAF levels, except for *L. idus* and *A. brama*, where Cu was not detected in their muscular tissue. This suggests species-specific differences in Cu uptake or metabolism. Mn bioaccumulation has a uniform distribution pattern among bioaccumulating species with little variation, with lower BSAF values compared to Fe and Cu. No specific pattern is observed in BCF and BSAF regardless of fish species or sampling sites. BCF data indicate that most species have the tendency to accumulate more Fe and Zn from water. BSAF, instead, shows that some species developed affinity for high levels of Fe accumulation, moderate levels of Cu, and lower levels of Mn accumulations, which, we consider to be the result of species-specific physiological mechanisms and habitat metal availability. The integrated BCF and BSAF show the vulnerability ranking of studied fish species. According to their values (3.90 and 3.74), results that *A. alburnus*, closely followed by *S. erythrophthalmus*, and *C. gibelio* have an increased susceptibility to metal bioaccumulation. *C. carpio* and *E. lucius* occupy an intermediate position (3.05), followed by *A. brama* (2.92), and *A. aspius* (1.55). *L. idus* (0.43) is less associated with metals accumulation capacity (Figure 7).

Vorkamp et al. (2016) report much higher Zn accumulation compared with our findings in *E. lucius* 558.179 vs. 1.03, *C. carpio* 381.14 vs. 1.19 *A. brama* 34.16 vs. 0.79 *C. gibelio* 318.69 s 0.87 [40]. In logarithmic terms, they obtained values ranging between 2.5 (*C. gibelio*) and 3 (*E. lucius*), while our findings fell within 0.0128 (*E. lucius*) and 0.1024 (*A. brama*). Starčević et al. (2025), in a study assessing metal content in Danube fish meat, discusses metal pollution trends over fifteen years [57], showing the period 2010–2012 a higher BCF interval of variation (1.484–9.68) for Fe [38,68] compared with our results, while for zinc, our results frame within the BCF interval (6.16–31 vs. 8.14) identified by their research [59,68].

### 4.4. Correlation and Multivariate Analysis of Heavy Metals Bioaccumulation and Proximate Composition in Fish Muscle Tissue

Testing the relationship between heavy metal bioaccumulation and the proximate composition of fish muscle tissue from the Babina–Cernovca area of the Danube Delta using Spearman correlation matrices (Figure 5), and principal component analyses (Figure 6) shows species-specific patterns and provides evidence for their associations.

In many fish species, essential metals (Fe, Zn, Cu) tend to correlate strongly and positively with protein (r > 0.70), suggesting that they have affinity for metalloproteins in the muscle tissue. The Zn–Cu and Fe–Se strong positive correlations in majority of muscle tissue of species studied show the possibility that they might use a common absorption pathway or have a common environmental source. The negative correlation Fe–Sb shows that these metals might be competing for absorption or show antagonistic biochemical interactions. Non-essential metals (Cr, Sb, Cd) correlated negatively to NFM, suggesting their potential inhibiting role in glycogen storage and/or carbohydrate metabolism. We found positive and strong correlations between protein and Zn, Cu, or Fe in benthivore species (*S. erythrophthalmus*, *L. idus*, *C. carpio*, and *C. gibelio*), while in piscivorous predator ones (*A. aspius* and *E. lucius*), there were stronger negative correlations with lipid content (Figure 5). PCA (Figure 6) shows the structuring of essential (Ti, Fe, Zn, Cu, Se) and non-essential/toxic (Cd, Sb, Hg, Sn) metals with proximate composition parameters. Function of feeding in Danube River Babina–Cernovca sector and distinct heavy metals association patterns are emphasized. The clustering patterns of heavy metals also give information about feed input sources, accumulation routes, or heavy metals metabolic roles [23]. The grouping of heavy metals usually suggests anthropogenic inputs mixed with environmental sources. The benthic-omnivorous feeding species (Table 1) show broader clustering of metals and composition traits, reflecting diverse pathways for heavy metals uptake. They exhibit a characteristic pattern characterized by constant positive loadings of Ti, Zn, Fe, Se, and predominantly of Cd, Cu, and protein on both PC1 and PC2 axes, showing connection with sedimental feed source, environmental conditions and mineral-binding mechanisms. The predominant Fe–Se grouping in benthic feeders predominantly aligns along the same PC2 axis, suggesting the importance of abiotic environment in their sediment feeding habits. In predatory and planktivorous species, heavy metals and proximate composition variables show a specific distribution, indicating selective dietary exposure. To *E. lucius*, *A. aspius*, piscivorous predators, corresponds to a pattern dominated by Cu, but also Ti. The lack of correlation consistency in this species underlines their distinct dietary pathways for metal uptake compared to planktivorous or benthivore species, where sediment contact amplifies exposure [69,70]. In *A. alburnus*, majority of identified metals are associated with PC1 and PC2. PCA output is confirmed by cluster correlation analyses (Figure 4 and Figure 5).

The constant presence of essential metals Ti, Fe, Zn, Cu, Se, Cd, Ag, Se in all studied fish species, even though they belong to three different groups according to their feeding habit, suggest the major influence of the shared environmental habitat located in Babina–Cernovca sector.

Future research is needed for extending monitoring efforts across multiple seasons and years to identify temporal variability in heavy metal bioaccumulation, which is needed for understanding long-term exposure risks and the influence of climatic and hydrological dynamics in the Danube Delta. Little research is focused on heavy metal human health risk assessment, and because of this reason we consider the integration of measured metal concentrations in fish muscle with dietary intake data from local communities to quantify potential food safety risks as a necessity. Such assessments should be explicitly aligned with the maximum levels established by Commission Regulation (EU) 2023/915 on contaminants in foodstuffs, which sets legally binding thresholds for cadmium, lead, and mercury in fish [71]. Molecular investigations of sentinel species such as *A. alburnus* and *C. gibelio* are needed to elucidate pathways of heavy metal uptake, detoxification, and storage, thereby supporting the development of biomarkers for early detection of contamination and strengthening biomonitoring strategies in ecologically sensitive areas. It should also be noted that, although unlikely to have influenced the validity of the results, a methodological limitation of this study is the lack of certified reference materials for the calibration of AAS in water analyses and XRF in solid matrices. Nevertheless, the accuracy of the determinations was safeguarded through the use of high-purity standard solutions in AAS and the application of the fundamental parameters method in XRF. This approach is further supported by the consistently low measurement errors obtained.

## 5. Conclusions

In Babina–Cernovca sector of the Danube Delta, relatively low heavy metal concentrations were identified in water, while in sediments and muscle of all fish studied, accumulation is observed. Except for Cd, which consistently exceeds regulatory safety thresholds in muscle tissue, and therefore represents a potential food safety concern for consumers; the other metals do not raise concerns. For Cu, Fe, and Mn, sediment (mud) is the primary reservoir, and for Zn and Fe, the role is played by water. The fish species exhibit different bioaccumulation capacity. According to integrated values of BCF and BSAF, *A. alburnus*, closely followed by *S. erythrophthalmus*, and *C. gibelio* showed the highest capacity, a fact that recommends these species to be used as sentinel species in biomonitoring studies meant to identify pollution levels in Danube River. From a food safety perspective, the high amount of Cd in muscle tissue highlights the necessity of routine monitoring to prevent human exposure through fish consumption, particularly in communities relying on local fish as a dietary habitual component. According to PCA, there are associations between identified heavy metals (Ti, Zn, Fe, Se, of Cd, Cu, and between these and protein), with strong associations between Fe and Se in benthic-omnivorous feeding species. This suggests characteristic feeding sources and physiological pathways. In piscivorous predators, the accumulation is mainly dominated by Cu and Ti, and the lack of strong correlations between heavy metals underlines the distinct feeding process in the same habitat. In *A. alburnus*, a special heavy metal accumulation pattern in relation to environment, feeding source, and species-specific physiological mechanisms was observed, resulting in the highest bioaccumulation capacity. The identification of Ti, Fe, Zn, Cu, Se, Cd, Ag, Se presence in all fish species could be the result of the influence of their common environmental habitat located in Babina–Cernovca sector. The study also emphasizes the value of combining univariate, bivariate, and multivariate statistical methods to clarify the factors influencing heavy metal bioaccumulation, but also the applied importance of food safety risk assessment and sustainable management of aquatic resources.

## Figures and Tables

**Table 1:** Scientific and common names with associated feeding behaviors of studied fish species [27].

No.	Scientific Name	Common Name	Feeding Behavior	
1	*Scardinius erythropthalmus* L.	Common rudd	Omnivorous, primarily benthivorous	* 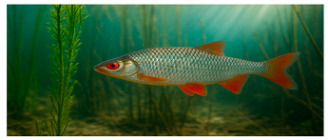 *
2	*Esox lucius* L.	Northern pike	Piscivorous predator	* 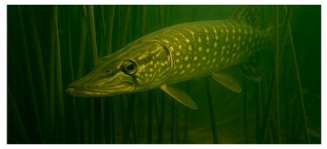 *
3	*Aspius aspius* L.	Asp	Piscivorous predator	* 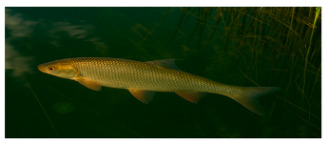 *
4	*Leuciscus idus* L.	Ide	Omnivorous primarily insectivorous	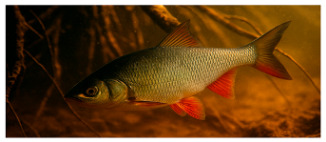
5	*Cyprinus carpio* L.	Common carp	Omnivorous and primarily benthivorous	* 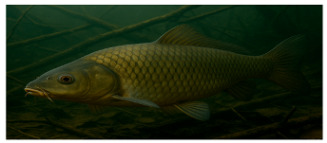 *
6	*Abramis brama* L.	Common bream	Omnivorous and primarily benthivorous	* 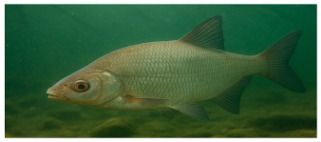 *
7	*Carassius gibelio* Bloch	Crucian carp	Omnivorous and primarily benthivorous	* 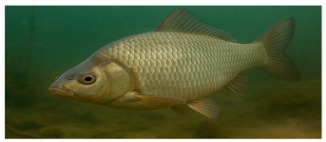 *
8	*Alburnus alburnus* L.	Common bleak	Planktivorous and insectivorous	* 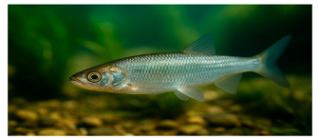 *

**Table 2:** The heavy metal concentrations (mg/L) in water and (mg/kg) mud from sampling points located in the Babina–Cernovca sector of the Danube Delta, collected in September 2023.

In Sampling Point P4	N	Cu	Zn	Fe	Mn
		X ± s_X_	X ± s_X_	X ± s_X_	X ± s_X_
P1 water	-	-	-	-	-
P2 water	-	-	-	-	-
P3 water	5	-	-	1.04 × 10^−6^ ± 2.63 × 10^−8^	-
P4 water	5	-	0.126 ± 0.002	0.021 ± 0.001	-
P5 water	-	-	-	-	-
P1 mud	5	0.071 ± 0.002	-	0.124 ± 0.003	-
P2 mud	5	0.054 ± 0.003	-	0.106 ± 0.002	0.070 ± 0.003
P3 mud	5	0.022 ± 0.001	-	0.226 ± 0.009	0.011 ± 0.35 × 10^−3^
P4 mud	5	0.036 ± 0.002	-	0.053 ± 0.001	0.072 ± 0.003
P5 mud	5	0.024 ± 0.001	-	0.059 ± 0.002	0.118 ± 0.002

N—number of replicates; X—average; s_X_—standard error (SE).

**Table 3:** The dry matter and proximate composition of the muscle tissue in fish species from the Babina–Cernovca sector of the Romanian Danube Delta, collected in September 2023 (% of fresh tissue—FT, and % of dry matter—DM).

Species	Dry Matter	Proximate Nutritional Components							
		Protein		Fat		Ash		Nitrogen-Free Matter	
		%FT	%DM	%FT	%DM	%FT	%DM	%FT	%DM
*Scardinius erythrophthalmus* L.	30.20 a ± 1.75	17.66 a ± 0.36	58.48 c ± 0.95	2.35 b ± 0.04	7.78 b ± 0.011	1.17 a ± 0.02	3.87 a ± 0.06	9.02 a ± 0.40	29.87 a ± 1.11
*Esox lucius* L.	24.52 bc ± 0.95	18.94 a ± 0.62	77.24 a ± 1.32	1.55 bc ± 0.07	6.32 b ± 0.12	1.35 a ± 0.08	5.51 a ± 0.15	2.68 c ± 0.09	10.93 c ± 0.25
*Aspius aspius* L.	24.14 bc ± 1.28	18.18 a ± 0.89	75.31 a ± 1.78	2.22 b ± 0.07	9.20 b ± 0.11	0.99 a ± 0.04	4.10 a ± 0.07	2.75 c ± 0.13	11.39 c ± 0.27
*Leuciscus idus* L.	24.39 bc ± 0.86	16.95 b ± 0.74	69.50 b ± 1.52	0.78 c ± 0.02	3.20 c ± 0.05	1.17 a ± 0.05	4.80 a ± 0.11	5.49 b ± 0.08	22.51 b ± 0.18
*Cyprinus carpio* L.	29.94 a ± 1.60	16.71 a ± 0.56	55.81 c ± 1.62	6.28 a ± 0.12	11.36 a ± 0.31	1.52 a ± 0.03	5.08 a ± 0.12	8.31 a ± 0.68	27.76 a ± 1.84
*Abramis brama* L.	26.87 b ± 0.79	17.64 a ± 0.41	65.65 b ± 0.94	1.47 bc ± 0.01	5.47 bc ± 0.03	1.64 a ± 0.05	6.10 a ± 0.14	6.12 a ± 0.32	22.78 b ± 1.02
*Carassius gibelio* Bloch	25.73 b ± 1.17	20.39 a ± 0.58	79.25 a ± 1.26	1.05 bc ± 0.06	4.08 bc ± 0.13	1.15 a ± 0.02	4.47 a ± 0.05	3.14 c ± 0.06	12.20 c ± 0.14
*Alburnus alburnus* L.	22.47 c ± 0.60	17.51 a ± 0.80	77.93 a ± 1.4	0.42 c ± 0.02	1.87 c ± 0.03	1.27 a ± 0.07	5.65 a ± 0.02	3.27 c ± 0.16	14.55 c ± 0.28

DM—dry matter; the differences between any two averages are significant, if their values are followed by different letters, or groups of different letters (*p* < 0.05).

**Table 4:** Heavy metal concentrations in the muscle tissue of fish species from the Babina–Cernovca sector of the Romanian Danube Delta, collected in September 2023 (mg/kg).

	*Scardinius erythrophthalmus* L.	*Esox* *lucius* L.	*Aspius * *aspius* L.	*Leuciscus idus* L.	*Cyprinus * *carpio* L.	*Abramis * *brama* L.	*Carassius * *gibelio* Bloch	*Alburnus* *alburnus* L.
Ti	0.29 a ± 0.012	0.24 a ± 0.007	0.28 a ± 0.006	0.27 a ± 0.013	0.22 a ± 0.010	0.23 a ± 0.006	0.19 a ± 0.009	0.30 a ± 0.011
Fe	1.80 a ± 0.076	1.71 a ± 0.060	0.25 b ± 0.015	0.18 b ± 0.006	1.71 a ± 0.052	1.79 a ± 0.040	1.98 a ± 0.117	2.00 a ± 0.048
Zn	0.20 a ± 0.005	0.13 a ± 0.006	0.18 a ± 0.005	0.16 ab ± 0.008	0.15 ab ± 0.011	0.10 b ± 0.003	0.11 b ± 0.003	0.19 a ± 0.008
Cr	0.66 a ± 0.028	0.55 ab ± 0.031	-	0.70 a ± 0.039	0.51 a ± 0.016	0.61 a ± 0.024	0.69 a ± 0.024	0.63 a ± 0.037
Cu	0.27 a ± 0.010	0.28 a ± 0.009	0.31 a ± 0.017	-	0.26 a ± 0.016	-	0.31 a ± 0.004	0.30 a ± 0.003
Sb	0.02 b ± 0.003	-	0.04 b ± 0.005	0.04 b ± 0.004	0.03 b ± 0.002	0.31 a ± 0.007	-	0.06 b ± 0.001
Mn	0.02 a ± 0.002	-	-	-	-	-	0.03 a ± 0.002	0.02 a ± 0.004
Cd	0.14 ab ± 0.006	0.20 a ± 0.007	0.14 ab ± 0.007	0.18 a ± 0.010	0.14 ab ± 0.005	0.15 ab ± 0.008	0.20 a ± 0.014	0.25 a ± 0.007
Ag	0.07 a ± 0.004	0.09 a ± 0.003	0.07 a ± 0.005	0.08 a ± 0.005	0.05 a ± 0.004	0.07 a ± 0.004	0.08 a ± 0.004	0.10 a ± 0.005
Ni	0.09 a ± 0.001	0.09 a ± 0.001	0.10 a ± 0.007	-	0.09 a ± 0.003	-	0.10 a ± 0.004	0.11 a ± 0.004
Se	0.99 b ± 0.012	2.88 a ± 0.060	3.16 a ± 0.094	3.03 a ± 0.123	2.24 a ± 0.084	1.87 a ± 0.127	1.92 a ± 0.048	1.99 a ± 0.099
V	-	-	0.05 a ± 0.002	-	-	-	0.05 a ± 0.002	-
Sn	-	0.04 a ± 0.004	-	-	0.03 a ± 0.002	0.04 a ± 0.001	0.04 a ± 0.002	0.05 a ± 0.002
Hg	-	-	-	-	-	0.07 a ± 0.004	-	-
As	-	2.04 a ± 0.045	-	2.05 a ± 0.062	2.07 a ± 0.076	1.99 a ± 0.097	-	-

The differences between any two averages are significant, if their values are followed by different letters, or groups of different letters (*p* < 0.05).

**Table 5:** Bioconcentration Factors (BCF) and log_10_ BCF in the muscle tissue of examined fish species relative to metal concentrations in mud from the Babina–Cernovca sector of the Danube Delta.

Species	BAC_Fe_						BAC_Zn_					
	P1	P2	P3	P4	log_10_	P5	P1	P2	P3	P4	log_10_	P5
a	-	-	-	1.59	0.2014	-	-	-	-	8.57	0.9330	-
b	-	-	-	1.03	0.0128	-	-	-	-	8.14	0.9106	-
c	-	-	-	1.43	0.1553	-	-	-	-	1.19	0.0755	-
d	-	-	-	1.27	0.1038	-	-	-	-	0.86	0.0655	-
e	-	-	-	1.19	0.0755	-	-	-	-	8.14	0.9106	-
f	-	-	-	0.79	0.1024	-	-	-	-	8.52	0.9304	-
g	-	-	-	0.87	0.0605	-	-	-	-	9.43	0.9745	-
h	-	-	-	1.51	0.1790	-	-	-	-	9.52	0.9786	-

BAI_Fe_—bioaccumulation index of iron (Fe); BAI_Zn_—bioaccumulation index of zinc (Zn); P1—sampling point 1; P2—sampling point 2; P3—sampling point 3; P4—sampling point 4; P5—sampling point 5; a—*Scardinius erythrophthalmus* L.; b—*Esox lucius* L.; c—*Aspius aspius* L.; d—*Leuciscus idus* L.; e—*Cyprinus carpio* L.; f—*Abramis brama* L.; g—*Carassius gibelio* Bloch; h—*Alburnus alburnus* L.

**Table 6:** Biota-Sediment Accumulation Factors (BSAF) and log_10_ BSAF for Cu in the muscle tissue of examined fish species relative to metal concentrations in mud from the Babina–Cernovca sector of the Danube Delta.

Species	BSAF						log_10_ BSAF					
	P1	P2	P3	P4	P5	X ± SE	P1	P2	P3	P4	P5	X ± SE
a	3.80	5.00	12.27	7.50	11.25	7.96 ± 1.93	0.5798	0.6990	1.0888	0.8751	1.0512	0.8588 ± 0.4690
b	3.94	5.19	12.73	7.78	11.67	8.26 ± 1.96	0.5955	0.7152	1.1048	0.8910	1.0671	0.8747 ± 0.4691
c	4.37	5.74	14.09	8.61	12.92	9.14 ± 2.07	0.6405	0.7589	1.1489	0.9350	1.1113	0.9189 ± 0.4688
d	-	-	-	-	-	-	-	-	-	-	-	-
e	3.66	4.81	11.82	7.22	10.83	7.66 ± 1.89	0.5635	0.6821	1.0726	0.8585	1.0346	0.8423 ± 0.4688
f	-	-	-	-	-	-	-	-	-	-	-	-
g	4.37	5.74	14.09	8.61	12.92	9.14 ± 2.07	0.6405	0.7589	1.1489	0.9350	1.1113	0.9189 ± 0.4688
h	4.23	5.56	13.64	8.33	12.50	8.85 ± 2.03	0.6263	0.7451	1.1348	0.9206	1.0969	0.9048 ± 0.4687

P1—sampling point 1; P2—sampling point 2; P3—sampling point 3; P4—sampling point 4; P5—sampling point 5; a—*Scardinius erythrophthalmus* L.; b—*Esox lucius* L.; c—*Aspius aspius* L.; d—*Leuciscus idus* L.; e—*Cyprinus carpio* L.; f—*Abramis brama* L.; g—*Carassius gibelio* Bloch; h—*Alburnus alburnus* L.

**Table 7:** Biota-Sediment Accumulation Factors (BSAF) and log_10_ BSAF for Fe in the muscle tissue of examined fish species relative to metal concentrations in mud from the Babina–Cernovca sector of the Danube Delta.

Species	BSAF						log_10_ BSAF					
	P1	P2	P3	P4	P5	X ± SE	P1	P2	P3	P4	P5	X ± SE
a	14.52	16.98	7.96	33.96	30.51	20.78 ± 3.32	1.1620	1.2299	0.9009	1.5310	1.4844	1.2616 ± 0.5065
b	13.79	16.13	7.57	32.26	28.98	19.74 ± 3.23	1.1396	1.2076	0.8791	1.5087	1.4621	1.2394 ± 0.5063
c	2.02	2.36	1.11	4.72	4.24	2.89 ± 1.23	0.3054	0.3729	0.0453	0.6739	0.6274	0.4050 ± 0.5059
d	1.45	1.70	0.80	3.40	3.05	2.08 ± 1.05	0.1614	0.2304	−0.0969	0.5315	0.4843	0.2621 ± 0.5059
e	13.79	16.13	7.57	32.26	28.98	19.74 ± 3.23	1.1396	1.2076	0.8791	1.5087	1.4621	1.2394 ± 0.5063
f	14.44	16.89	7.92	33.77	30.34	20.67 ± 3.31	1.1596	1.2276	0.8987	1.5285	1.4820	1.2593 ± 0.5064
g	15.97	18.68	8.76	37.36	33.56	22.86 ± 3.48	1.2033	1.2714	0.9425	1.5724	1.5258	1.3031 ± 0.5064
h	16.13	18.87	8.85	37.74	33.90	23.09 ± 3.50	1.2076	1.2758	0.9469	1.5768	1.5302	1.3075 ± 0.5064

P1—sampling point 1; P2—sampling point 2; P3—sampling point 3; P4—sampling point 4; P5—sampling point 5; a—*Scardinius erythrophthalmus* L.; b—*Esox lucius* L.; c—*Aspius aspius* L.; d—*Leuciscus idus* L.; e—*Cyprinus carpio* L.; f—*Abramis brama* L.; g—*Carassius gibelio* Bloch; h—*Alburnus alburnus* L.

**Table 8:** Biota-Sediment Accumulation Factors (BSAF) and log_10_ BSAF for Mn in the muscle tissue of examined fish species relative to metal concentrations in mud from the Babina–Cernovca sector of the Danube Delta.

Species	BSAF						log_10_ BSAF					
	P1	P2	P3	P4	P5	X ± SE	P1	P2	P3	P4	P5	X ± SE
a	-	0.29	1.82	0.28	0.17	0.64 ± 0.88	-	0.538	0.260	0.553	0.770	0.5303 ± 0.4572
b	-	-	-	-	-	-	-	-	-	-	-	-
c	-	-	-	-	-	-	-	-	-	-	-	-
d	-	-	-	-	-	-	-	-	-	-	-	-
e	-	-	-	-	-	-	-	-	-	-	-	-
f	-	-	-	-	-	-	-	-	-	-	-	-
g	-	0.43	2.73	0.42	0.25	0.95 ± 1.08	-	0.367	0.436	0.377	0.602	0.4455 ± 0.3297
h	-	0.29	1.82	0.28	0.17	0.64 ± 0.88	-	0.538	0.260	0.553	0.770	0.5303 ± 0.4572

P1—sampling point 1; P2—sampling point 2; P3—sampling point 3; P4—sampling point 4; P5—sampling point 5; a—*Scardinius erythrophthalmus* L.; b—*Esox lucius* L.; c—*Aspius aspius* L.; d—*Leuciscus idus* L.; e—*Cyprinus carpio* L.; f—*Abramis brama* L.; g—*Carassius gibelio* Bloch; h—*Alburnus alburnus* L.

**Table 9:** Total variance and Eigenvalues in PC plans for fish species from Babina–Cernovca sector of the Danube Delta.

Species	PC	Eigenvalue	%Total	Species	PC	Eigenvalue	%Total
a—*S. erythrophthalmus*	PC1	6.429895	40.18685	b—*E. lucius*	PC1	6.676482	41.72801
	PC2	4.906004	30.66252		PC2	5.884697	36.77936
	Total	11.3359	70.84937		Total	12.56118	78.50737
c—*A. aspius*	PC1	6.602411	44.01607	d—*L. idus*	PC1	6.389428	45.63877
	PC2	4.642895	30.95264		PC2	4.093006	29.23575
	Total	11.24531	74.96871		Total	10.48243	74.87452
e—*C. carpio*	PC1	6.946362	40.86095	f—*A. brama*	PC1	6.123379	38.27112
	PC2	5.845441	34.38495		PC2	5.561175	34.75734
	Total	12.7918	75.2459		Total	11.68455	73.02846
g—*C. gibelio*	PC1	6.897949	40.57617	h—*A. alburnus*	PC1	6.370362	37.47272
	PC2	5.440019	32.00011		PC2	5.652739	33.25140
	Total	12.33797	72.57628		Total	12.0231	70.72412

The a–h labeling signifies the correspondence with figures a–h emphasizing the Principal Component Analysis (PCA) biplots.

## Data Availability

The raw data supporting the conclusions of this article will be made available by the authors on request.

## Abbreviations

The following abbreviations are used in this manuscript:

ANOVA	Analysis of variance
PCA	Principal Components Analysis
UNESCO	United Nations Educational, Scientific and Cultural Organization
RAMSAR	Convention on Wetlands of International Importance especially as Waterfowl Habitat
DM	Dry matter
P	Protein
F	Fat
A	Ash
NFM	Nitrogen-free matter
BCF	Bioconcentration Factor
BSAF	Biota-Sediment Accumulation Factor
PC	Principal Component
KMO	Keiser–Meyer–Olkin
ARBDD	Delta Biosphere Reserve Authority
N	Number of replicates
X	Average
s_X_	Standard error
R_S_	Spearman coefficient of correlation
